# Anemia among Medical Students from Jakarta: Indonesia—Iron Deficiency or Carrier Thalassemia?

**DOI:** 10.1155/2024/4215439

**Published:** 2024-04-12

**Authors:** Raditya Wratsangka, Endrico Xavierees Tungka, Aditya Krishna Murthi, Soegianto Ali, Ita Margaretha Nainggolan, Edhyana Sahiratmadja

**Affiliations:** ^1^Department of Obstetrics and Gynecology, Faculty of Medicine, Universitas Trisakti, West Jakarta, Indonesia; ^2^Department of Biochemistry, Faculty of Medicine, Universitas Trisakti, West Jakarta, Indonesia; ^3^Department of Medical Physiology, Faculty of Medicine, Universitas Trisakti, West Jakarta, Indonesia; ^4^Department of Medical Biology, Biomedical Science Study Program, School of Medicine and Health Sciences, Atma Jaya Catholic University of Indonesia, South Jakarta, Indonesia; ^5^Biomedical Science Study Program, School of Medicine and Health Sciences, Atma Jaya Catholic University of Indonesia, South Jakarta, Indonesia; ^6^Eijkman Research Center for Molecular Biology, National Research and Innovation Agency, Bogor, Indonesia; ^7^Department of Biomedical Sciences, Faculty of Medicine, Universitas Padjadjaran, Bandung, Indonesia

## Abstract

**Background:**

Anemia, a global health concern, affects one-fourth of the global population, particularly women. In Indonesia, its prevalence is 23.7%, with 32.0% among 15-24 year-olds. Factors include poor nutrition, infectious diseases, chronic diseases, inherited disorders, and inadequate healthcare access. This study aimed to investigate anemia prevalence and its etiology among medical students from Jakarta.

**Methods:**

This study was a descriptive research with a cross-sectional approach. Undergraduate students aged 18–23 years old were selected and consented to participate by a consecutive nonrandom sampling methods. Laboratory blood data were evaluated (including Hb, MCV, MCH, HbA_2_, and ferritin levels) and DNA was isolated to confirm the type of thalassemia carrier.

**Results:**

In total, 140 medical students, mainly female, were recruited. Anemia was found in 13.6% (11.4% had low MCV and/or MCH), and 16.5% had low MCV and/or MCH without anemia. Hb electrophoresis revealed high HbA_2_ values, suggesting the HbE variant (2.1%), and *β*-thalassemia carrier (0.7%). DNA analysis confirmed the cd26 mutation and heterozygous IVS1nt5. Among those without anemia, 5% had *α-deletion*, while in the group with anemia, 1.4% had *α-deletion* (with coexistent IDA), 3.6% had *α-deletion*, and 0.7% had *β-mutation*.

**Conclusion:**

DNA analysis can identify specific mutations associated with alpha-thalassemia, distinguishing between iron deficiency anemia and the alpha-thalassemia trait. Thalassemia screening should involve low MCV and/or MCH values as the first step (stage 1), followed by Hb analysis (stage 2) and DNA analysis (stage 3). In common areas, a combination of Hb and DNA testing is best. However, healthcare professionals must diagnose and treat thalassemia, as proper management relies on accurately identifying the underlying condition.

## 1. Introduction

Anemia is a worldwide public health issue, affecting both developed and developing nations. Anemia is also the third leading cause of years with lived disability (YLDs) [1]. One-fourth of the global population is estimated to be anemic, with cases increasing rapidly in women. Over half a billion women between the ages of 15 and 49 made up the global anemia prevalence in 2019, including women of reproductive age (WRA; 29.9%), pregnant women (36.5%), and nonpregnant WRA (29.6%), with sub-Saharan Africa and South Asia having the most significant rates [1]. In 2021, there will be an increase of 420 million cases over three decades [1, 2]. Data from Indonesia revealed that the prevalence of anemia in Indonesia was 23.7% [3]. Additionally, the prevalence of anemia among individuals aged 15–24 years was 32.0%.

The high prevalence of anemia among young adults in Indonesia may differ among regions and population subgroups and is likely due to a combination of factors, including poor nutrition, infectious diseases, chronic diseases, inherited disorders (sickle cell anemia, thalassemia), and inadequate access to healthcare [4, 5]. Interestingly, recent evidence has shown that the awareness programs and laboratory examinations have identified more thalassemia carriers, minors, or traits among young adults [6].

Thalassemia carriers have a small red blood size (microcytic), as shown in low MCV values, and/or less red blood cell color (hypochromic), as shown in low MCH values. Thalassemia carrier does not necessarily have low Hb. Anemia and thalassemia can cause low levels of hemoglobin in the blood, leading to low levels of red blood cells and decreased oxygen delivery to tissues [7]. Thalassemia is one condition that can cause anemia; however, thalassemia minor can be found without anemia [7]. The two conditions share some similarities, but have some essential differences.

Thalassemia is an inherited autosomal recessive gene disorder caused by a decreased or absent production of one or two types of globin chains and classified according to the particular globin chain affected, such as *α*-globin and *β*-globin. Thalassemia is also a significant health issue in Indonesia, particularly among young adults [8, 9]. Indonesia is located along the “Thalassemia Belt,” a geographic region that stretches from the Mediterranean to Southeast Asia, where the prevalence of thalassemia is highest [10–12].

Medical students, as part of the young adult population, are obliged to study various diseases, including anemia and thalassemia in their courses on genetics, hematology, and other related topics. Medical students' knowledge, attitudes, and practice towards thalassemia are likely to be shaped by various factors, including their academic and personal backgrounds, their exposure to patients with thalassemia, and their understanding of the social and cultural contexts in which the disease occurs. Medical students who look physically fit may not even know their status as anemia and carriers of thalassemia traits [13–16]. A study in Malaysia involving 400 undergraduate medical students has reported that 14.5% students had hypochromic microcytic red cell indices, 11% showed thalassemia red cell indices, and only 3.5% had iron deficiency (IDA) red cell index which were finally confirmed by serum iron/TIBC analysis [17].

Therefore, it is interesting to also explore the etiology of anemia among students in Indonesia compared to the neighboring country of Malaysia. The study aimed to explore the anemia prevalence and etiology among medical students from Jakarta.

## 2. Materials and Methods

### 2.1. Study Design

This study was descriptive research with a cross-sectional approach to explore the anemia prevalence among medical students and the etiology of the anemia. Laboratory blood data were evaluated (including Hb, MCV, MCH, HbA_2_, and ferritin levels) and DNA was isolated to confirm the type of thalassemia carrier (trait). The study was approved by the Research Ethics Committee of the Faculty of Medicine, Universitas Trisakti (No. 011/KER/FK/IV/2021).

### 2.2. Subjects

Undergraduate students of the Faculty of Medicine, Universitas Trisakti, aged 18–23 were selected and consented to participate by a consecutive nonrandom sampling methods. Those who had a history of chronic disease and had received blood transfusions in the past three months were excluded from this study.

### 2.3. Complete Blood Count and HbA_2_ Measurement

Venous blood sampling was collected in a 3 ml EDTA tube to be checked further for a complete blood count (CBC), followed by ferritin measurement and HbA_2_ determination by highperformance liquid chromatography (Bio-Rad). CBC was analyzed using the flow cytometry method using a semiconductor laser (Sysmex-XN®); ferritin measurement was analyzed using the chemiluminescent magnetic microparticle immunoassay (CMIA) method (Architect® i2000). The definition of anemia is according to WHO standards based on sex (men <13 g/dL and women <12 g/dL). The red blood cell (RBC) indices were then analyzed based on a guide from the Ministry of Health, Republic of Indonesia, MCV< 80 fL and MCH <27 pg values which were used as initial thalassemia screening. Furthermore, Hb analysis data were classified based on HbA_2_ fraction values according to the protocol of the manufacturer (Bio-Rad) where <2.0% suggested for *α*-thalassemia carriers; 2.0–3.5% as normal value; >3.5% was indicated as *β*-thalassemia carriers; and very high HbA_2_ level peak shown indicated HbE carriers. Hereafter, subjects who had normal HbA_2_ but had low/normal Hb and/or low MCV and/or low MCH were examined further for DNA analysis. Those with low MCV and/or MCH and with normal HbA_2_ value (2–3.5%) were further confirmed with DNA examination to distinguish the etiology of anemia whether due to iron deficiency or alpha-thalassemia carrier. The students were also checked for their ferritin levels to determine whether anemia was due to iron deficiency.

### 2.4. DNA Genotyping Identification

Subjects with low MCV and/or MCH, low and normal Hb, and low and normal ferritin were identified by the level of HbA_2_ and subsequently confirmed their genotype by DNA analysis (*N* = 39). Due to the limited availability of examination kits, DNA tests were carried out on 30 of 39 subjects, whose selection was done by simple random sampling.

We used the *α*-Globin StripAssay® (Ref. 4–160, ViennaLab Diagnostics GmbH) to detect 21 common *α*-globin mutations in separated teststrips. The procedure includes three steps: [1] DNA isolation, [2] PCR amplification using biotinylated primers, [3] hybridization of amplification products to teststrips containing allele-specific oligonucleotide probes immobilized as an array of parallel line. The bound biotinylated sequences on specific wild types and mutants are detected using streptavidin-alkaline phosphatase and color substrates. We prepared three separate PCR mixtures (A1, A2, and B), following both PCR conditions and hybridization according to the manufacturer's instructions. We analyzed the hybridization results by comparing the line with the provided CollectorTM sheet. In heterozygous conditions, both mutated bands and normal bands will appear. In homozygous mutations, only the positive band is colored. If no mutations are contained in the StripAssay panel, only PCR and normal controls are colored. Furthermore, to identify *β*-globin gene mutations, we used the *β*-Globin StripAssay® SEA (Ref. 4–150, Vienna Lab Diagnostics GmbH). The assay covers 22 common *β*-globin mutations in one strip only. Unlike the assay for *α*-thalassemia, the assay for *β*-thalassemia was only in one PCR mixture and followed the manufacturer's instructions. The hybridization procedure and analysis of test strips results were the same as in *α*-globin gene mutation detection.

## 3. Result

In total, 140 medical students were recruited, primarily female (69.3%; *n* = 97), with a median age of 20 (range 18–26 years). The level of Hb in this study is 12.7 g/dL among female and 15.7 g/dL among male students. Anemia was found in 13.6% (*n* = 19) of the female students (Table 1). Interestingly, 16.5% of students (*n* = 23; 18 females and 5 males) were not anemic but had low MCV and/or low MCH. These students were subjected to the next level of the thalassemia screening program as indicated by the Ministry of Health. In addition, 16 (11.4%) students who were anemic and had low MCV and/or MCH were examined further to determine the etiology of anemia.

In our study, the students were examined by Hb electrophoresis (level 2), resulting in a very high HbA_2_ value (>13%), suggesting an HbE variant (2.1%; *n* = 3); and a high HbA_2_ value (>4% to 13%), indicating *β*-thalassemia carrier (0.7%; *n* = 1); that was confirmed by DNA analysis (level 3), resulting in cd26 mutation and heterozygous IVS1nt5, respectively (Table 2).

Among those without anemia (*n* = 15), 5% (*n* = 7) had *α*-deletion (Table 3). Furthermore, in a group with anemia (*n* = 14), *α*-deletion (with coexistent IDA) was detected in 1.4% (*n* = 2), *α*-deletion 3.6% (*n* = 5), and *β*-mutation in 0.7% (*n* = 1).

## 4. Discussion

Our study has shown that among medical students aged 18 to 23 years old, anemia has been detected in 13.6% (*n* = 19) students, all females. Interestingly, 16.5% of students (18 females and 5 males) who are not anemic have low MCV and/or MCH. All these students have been examined for ferritin, Hb electrophoresis, and DNA genotyping for alpha and beta-globin to explore the etiology of anemia.

If a female is experiencing mild anemia but is otherwise in good physical condition, it is essential to consider the potential causes and appropriate management strategies for her condition. Anemia, characterized by a decrease in the number of red blood cells or the amount of hemoglobin in the blood, can lead to various symptoms, including fatigue, weakness, pale skin, and shortness of breath, among others. However, mild cases may have minimal or no symptoms, especially if the individual is generally healthy and physically active [18].

Medical students who have a middle-class socio-economic status and are knowledgeable about anemia and thalassemia should be aware of the effects of poor nutrition and inadequate dietary iron intake. Not consuming enough iron-rich foods (including red meat, poultry, fish, beans, lentils, tofu, fortified cereals, and green leafy vegetables) in their diet can lead to IDA, which is assumed to be low [19–21]. However, the results showed that 13.6% (*n* = 19) had anemia and additionally, 16.5% (*n* = 23) were not anemic but had low MCV/MCH.

Anemia characterized by a normal MCV/MCH is known as normocytic or normochromic anemia. This form of anemia is distinguished by red blood cells that are of average size (normocytic) and contain a normal amount of hemoglobin (normochromic). The causes can vary and encompass: acute blood lost, chronic diseases, aplastic anemia, hemolytic anemias, anemia of persistent disease, renal anemia, bone marrow disorders, and endocrine problems such as hypothyroidism or hypopituitarism. Diagnosis of the specific cause of normocytic, normochromic anemia involves a thorough medical history, physical examination, and a series of laboratory tests beyond just the complete blood count (CBC) that measures MCV and MCH, such as reticulocyte count, serum iron levels, ferritin, total iron-binding capacity (TIBC), vitamin B12, and folate levels, as well as tests for renal function and possible bone marrow examination.

Patients with low MCV/MCH values typically exhibit signs of microcytic and hypochromic anemias, which are often associated with iron deficiency or disorders of hemoglobin synthesis like thalassemia. However, if ferritin levels are normal and there are no mutations in the DNA for alpha- and beta-globin genes (which would indicate thalassemia), other potential causes could include: anemia of chronic disease, lead poisoning, sideroblastic anemia, copper deficiency, rare hemoglobin variants, and chronic kidney disease. It is important to note that the diagnosis of anemia and its underlying cause is complex and requires a comprehensive approach, including detailed patient history, physical examination, and a broader panel of laboratory tests beyond MCV, MCH, and ferritin levels. Consulting with a hematologist may be beneficial for patients with unexplained anemia.

Indonesia, located in the thalassemia belt area, has many thalassemia careers in Southeast Asia, making anemia in young adulthood a significant concern. However, no result of non-severe thalassemia disease has been found among medical students in this study. Anemia in young adults can have other causes, such as hemoglobin abnormalities or hemoglobinopathy, as the most common causes. Efforts to detect early thalassemia careers have not been carried out systematically. Around 3.0–10.0% of the Indonesian population carry *β*-thalassemia (*β*-thal) and 2.6–11.0% of the population carry *α*-thalassemia (*α*-thal), with the highest incidence rates reported in regions such as BAli, East Nusa Tenggara, and Maluku [9]. It is estimated that around 2500 babies are born with *β*-thal major (*β*-TM) each year [9].

### 4.1. Iron-Deficiency Anemia or Thalassemia Carrier?

Further analysis with Hb electrophoresis has shown that it is difficult to distinguish between IDA and alpha-thalassemia carriers. Both conditions can lead to microcytic (small red blood cells) and hypochromic (pale) red blood cells, making it necessary to perform additional tests, such as DNA examination, to make a definitive diagnosis. In restricted facilities, MCV <80 fl and/or MCH <27 pg is the optimal first-round mass screening approach for *β*-thalassemia carriers. Gradually implement Hb electrophoresis, and DNA analysis should be done in many regions to confirm mutations for accurate carrier detection wherever possible [22]. Since the machine BioRad Variant that we used has suggested cut off value of 4% to diagnose beta-thalassemia trait, our study has used lower cutoff (HbA_2_ > 3.5%). The cutoff value of hemoglobin A_2_ (HbA_2_) to diagnose beta-thalassemia trait can vary slightly depending on the laboratory and the population being studied. However, in general, a level of HbA_2_ greater than 3.5% is often considered suggestive of beta-thalassemia trait. This is typically confirmed through additional testing, such as hemoglobin electrophoresis or genetic testing, to definitively diagnose beta-thalassemia trait. It is important to note that interpretation of HbA_2_ levels should be done in conjunction with clinical findings and other laboratory tests for a comprehensive diagnosis [23–26].

Subjects with HbA_2_ level at > 13% could be diagnosed as Hb E trait and also possible alpha-thalassemia disease, something that we should be aware of. Since we did not examine for alpha-thalassemia deletion and/or mutation, this cannot be well concluded. To distinguish between Hemoglobin E (HbE) trait and alpha-thalassemia trait, one must comprehend the genetic and hematological distinctions that exist between these two disorders. Both are types of thalassemia, hereditary blood illnesses characterized by decreased hemoglobin production, resulting in anemia. Colaco [25] discusses the genetic and diagnostic implications of borderline HbA_2_ levels, which can be caused by various molecular defects. Nevertheless, they vary in terms of the particular globin chains that are impacted and their geographical distribution. In order to definitively distinguish between the HbE trait and alpha-thalassemia trait, medical professionals frequently utilise a combination of hematological tests, including a complete blood count (CBC), hemoglobin electrophoresis, and occasionally DNA analysis to identify genetic abnormalities or deletions. Individuals who are suspected of having these disorders should seek consultation with a healthcare professional or a hematologist in order to obtain an accurate diagnosis and appropriate management.

The prevalence of anemia among medical students varies depending on numerous factors, but being knowledgeable in medical science and having a sufficient socioeconomic background might be causes by other than IDA. A study of medical students from other parts of Indonesia has shown that only 7.9% are anemic, similar to other studies in Malaysia. The low prevalence of anemia is also found in Malaysia among Malay (6.6%), Chinese (6.9%), and Indian (26%) [17]. Moreover, among medical students, anemia was observed in 33.4% of Pakistani students [27], in 28.3% of Indian students from Bangalore [28], and 52.7% in Nepal [29].

Some studies mainly use CBCs from venous blood, while others use finger-pricking methods. These methods can produce different outcomes due to differences in hemoglobin measurement [30–32]. Venous blood is preferred for precise and accurate measurements of hemoglobin levels and other blood parameters. Fingerstick tests are used for point-of-care testing, rapid screenings, and immediate findings. However, they may need to be more precise in detecting blood disorders or making life-saving medical decisions. Medical experts often use venous blood testing for verification and more precise evaluation when clinical concerns arise.

Medical students, like many other young adults, can be susceptible to anemia due to dietary habits, lifestyle choices, and stress. The high cost of medical education at least indicates that their socio-economic status is quite good, however, they often have demanding schedules and face high levels of stress, which can contribute to poor eating habits, irregular meals, and inadequate nutrition. Moreover, prolonged study periods and a lack of physical activity can impact their health. Promoting healthy lifestyles, providing nutritious food options, encouraging physical activity, and raising awareness about maintaining good health can help mitigate the risk of anemia among medical students [33, 34].

Anemia affects more women than men, especially those of reproductive age, and is more prevalent in areas with high iron deficiency or parasite diseases. Female adolescents have lower hemoglobin levels than male adolescents, with anemia prevalence in women being 33.7% compared to 11.3% in men during reproductive years [1, 35]. It was largely due to factors like menstrual blood loss in females and hormonal differences between the genders. Menstrual blood loss indeed contributes to lower hemoglobin levels in females compared to males. Testosterone, higher in males, does not directly impact hemoglobin levels but may reflect differences in overall health. However, it is essential to note that lower hemoglobin levels in females are not always indicative of underlying health issues; they can be a natural consequence of menstruation. Kannan [36] reported a high prevalence of anemia among female medical students, with a correlation to menstrual abnormalities and nutritional habits. Alsheikh [37] found a high prevalence of iron deficiency anemia among Saudi female medical students, but no significant correlation with background, gynecological history, or dietary habits.

Male adolescents have higher testosterone levels, which typically rise during puberty, contributing to physical changes like muscle growth, deepening of the voice, and the development of male sexual characteristics. While testosterone itself doesn't directly cause anemia, certain conditions associated with hormonal imbalances or other health issues might impact hemoglobin levels in males. Anemia in male adolescents can occur due to various reasons unrelated to testosterone levels. Iron deficiency, chronic diseases, certain genetic conditions affecting red blood cell production, or nutritional deficiencies can lead to anemia in males [38, 39].

Thalassemia is a genetic blood disorder that affects hemoglobin synthesis. Thalassemia trait refers to thalassemia carriers with one defective and one normal hemoglobin gene. This defect results in reduced production of normal hemoglobin, leading to smaller and paler red blood cells (low MCV/MCH) but with normal overall hemoglobin levels (normal Hb). Microcytic anemia can occur if the mean corpuscular volume (MCV) and mean corpuscular hemoglobin (MCH) are both low. Iron deficiency, which can result from insufficient food intake, malabsorption, chronic or persistent blood loss, or increased demand for iron during pregnancy, is the most prevalent type of microcytic anemia. Other causes of microcytic anemia include hemoglobinopathy and sideroblastic anemia, a rare condition where the bone marrow produces abnormal red blood cells [40–43].

### 4.2. Difficulty to Distinguish IDA and Alpha-Thalassemia Trait

Iron deficiency anemia (IDA) and alpha-thalassemia trait (TT) are common conditions of microcytic hypochromic anemia (MHA). Distinguishing IDA and the alpha-thalassemia trait can be challenging due to their similar symptoms and laboratory findings. Iron therapy, usually oral or intravenous iron supplementation, is the primary treatment for IDA. Monitoring the patient's response to iron therapy is crucial [44]. Close monitoring of the patient's response to iron therapy is crucial during the diagnostic trial. Self-administering iron supplements without proper diagnosis and supervision is not advisable, as excessive iron intake can have adverse health effects [45]. Alpha-thalassemia is a genetic condition affecting alpha-globin chain production in hemoglobin. Alpha-thalassemia trait carriers typically have mild or no symptoms, and their hemoglobin levels are usually within the normal range. The alpha-thalassemia trait does not require iron therapy, as it is unrelated to an iron deficiency. To differentiate between IDA and the alpha-thalassemia trait, healthcare providers often use various diagnostic tools, including blood tests, hemoglobin electrophoresis, and DNA analysis [46]. Hemoglobin electrophoresis can help identify the specific type of hemoglobin and reveal the presence of alpha-thalassemia. DNA analysis, such as polymerase chain reaction (PCR) or genetic testing, can definitively diagnose of alpha-thalassemia by detecting specific genetic mutations associated with this condition. If there is uncertainty in the diagnosis or a need for confirmation, further DNA examination can be valuable in determining the presence of alpha-thalassemia or other hemoglobinopathies. Both IDA and the alpha-thalassemia trait can coexist, making a precise diagnosis even more important.

### 4.3. Limitations of the Study

The study's limitations include limited availability of examination kits for subjects with anemia and low MCV/MCH, and no iron therapy was given to those suspected of IDA. Iron therapy can be used as a diagnostic tool to help distinguish between IDA and the alpha-thalassemia trait, especially when there is uncertainty in the diagnosis. The other limitation of this study was the small number of respondents, with a female majority. There are parameters related to RBC that are lower in females compared to males. We also did not ask in detail about the pattern of menstrual bleeding, whether it is heavy bleeding, therefore, we could not analyse the relation between menstrual bleeding and low ferritin.

## 5. Conclusion

Thalassemia is a genetic blood disorder characterized by abnormal hemoglobin production that results in reduced production of hemoglobin and red blood cells, leading to anemia. Low MCV and/or MCH (stage 1) are common indicators observed in thalassemia, but hemoglobin electrophoresis (stage 2) and DNA genotyping (stage 3) are two essential tests needed in diagnosing thalassemia. Genetic testing can identify specific mutations or changes in the genes responsible for hemoglobin production to confirm the presence of thalassemia and determine its type (alpha thalassemia or beta-thalassemia) and severity. DNA analysis can also distinguish between iron deficiency anemia and the alpha-thalassemia trait, which has many similarities. In places where thalassemia is common, combining Hb analysis and DNA testing as the second and third stage tests is the best technique to determine a person's career status. However, a healthcare professional must make the diagnosis and treatment plan, as proper management depends on accurately identifying the underlying condition.

## Figures and Tables

**Table 1:** Hemoglobin level based on gender and MCV and/or MCH level in medical students (*n* = 140).

	Level of hemoglobin		
	Not anemia *n* (%)	Anemia *n* (%)	Total *n* (%)
Male	43 (30.7)	0	43 (30.7)
MCV and/or MCH			
(i) Normal	38 (27.1)	0	38 (27.1)
(ii) Low	5 (3.6)^*∗*^	0	5 (3.6)
Female	78 (55.7)	19 (13.6)	97 (69.3)
MCV and/or MCH			
(i) Normal	60 (42.9)	3 (2.1)	63 (45.0)
(ii) Low	18 (12.9)^*∗*^	16 (11.4)^*∗∗*^	34 (24.3)

*Note*. Anemia Hb: <13.0 g/dL (male); <12.0 g/dL (female). Low MCV: <80 fL. Low MCH: <27 pg. ^*∗*^Not anemia but with low MCV and/or MCH, subjected for further examination. ^*∗∗*^Anemia and were further examined to explore the etiology of anemia.

**Table 2:** Complete blood count examination (level 1) followed by Hb variant A_2_ (level 2) and DNA confirmation (level 3) among medical students from a private university in Jakarta (*n* = 140).

Level 1		Level 2		
MCV and/or MCH	Hb level	HbA_2_		
		2–3.5%	>3.5%	>>>13%
		*n* (%)	*n* (%)	*n* (%)
Low (*n* = 39)	Not anemia	20 (14.3)^*∗∗∗*^	0	3 (2.1)^*∗*^
	Anemia	15 (10.7)^*∗∗∗∗*^	1 (0.7)^*∗∗*^	0

Normal (*n* = 101)	Not anemia	98 (70.0)	0	0
	Anemia	3 (2.1)	0	0

*Note*. ^*∗*^Cd26 (G > A) heterozygote; ^*∗∗*^IVS1nt5 heterozygote; ^*∗∗∗*^further DNA analysis needed whether iron deficiency anemia or alpha. Thalassemia carrier. ^*∗∗∗∗*^Need iron therapy first.

**Table 3:** Evaluation of ferritin among students (*n* = 30) with low MCV and/or MCH (level 1) in relation to hemoglobin level, HbA_2_ (level 2) and DNA genotype (level 3).

Level 1 (low MCV/MCH) *N* = 30	Level 2 Hb electrophoresis (HbA_2_)			Level 3 DNA genotype
	2–3.5	>3.5	>>>	
(i) Not anemia (*n* = 16)				
(1) Ferritin low (*n* = 1)	1			Βeta (*β*): IVS1-nt5 [G > C] heterozygote + coexistent IDA^*∗∗∗*)^
(2) Ferritin normal (*n* = 15)^*∗*)^			3	Confirmed HbE: codon 26 [G > A] heterozygote
	4			No alpha (*α*) deletion or mutation found, need further exploration
	4			Alpha (*α*): single *α*-globin gene deletion 3.7 kb type heterozygote
	3			Alpha (*α*): double *α*-globin gene deletion SEA type heterozygote
(ii) Anemia (*n* = 14)				
(1) Ferritin low (*n* = 6)	4			No-*α* deletion or mutation; suggesting IDA
	1			Alpha (*α*): 3.7 kb triplicated *α* gene heterozygote + coexistent IDA^*∗∗∗*)^
	1			Alpha (*α*): *α*2 cd 59 [GGC > GAC] heterozygote + coexistent IDA^*∗∗∗*)^
(2) Ferritin normal (*n* = 8)^*∗∗*)^				
	3			Alpha (*α*): double *α*-globin gene deletion SEA type heterozygote
	1			Alpha (*α*): *α*2 cd 142 [TAA > CAA] (Hb constant spring) heterozygote
	1			Alpha (*α*): *α*2 cd 59 [GGC > GAC] heterozygote
		1		Beta (*β*): IVS1-nt5 [G > C] heterozygote

*Note.* Anemia was designated as Hb < 12.0 g/dL for females and Hb < 13.0 g/dL for males. ^*∗*)^One sample DNA examination failed. ^*∗∗*)^Two samples DNA examination failed. ^*∗∗∗*)^Point of interest; IDA with coexistence confirmed alpha ($\overset{´}{\alpha }$).

## Data Availability

The SPSS data used to support the findings of this study have been deposited in the Google drive repository https://drive.Google.com/drive/folders/1gjg7998MjKi9H2NVr-HoG4EgurO8FZC2?usp=drive_link (SPSS data format file and Excel format file).
